# Commissioning and performance evaluation of RadCalc for the Elekta unity MRI‐linac

**DOI:** 10.1002/acm2.12760

**Published:** 2019-11-13

**Authors:** Stephen A. Graves, Jeffrey E. Snyder, Amanda Boczkowski, Joël St‐Aubin, Dongxu Wang, Sridhar Yaddanapudi, Daniel E. Hyer

**Affiliations:** ^1^ Department of Radiation Oncology University of Iowa Iowa City IA USA; ^2^ Memorial Sloan Kettering Cancer Center West Harrison NY USA

**Keywords:** dose calculation, Elekta Unity, MRI‐Linac, RadCalc

## Abstract

Recent availability of MRI‐guided linear accelerators has introduced a number of clinical challenges, particularly in the context of online plan adaptation. Paramount among these is verification of plan quality prior to patient treatment. Currently, there are no commercial products available for monitor unit verification that fully support the newly FDA cleared Elekta Unity 1.5 T MRI‐linac. In this work, we investigate the accuracy and precision of RadCalc for this purpose, which is a software package that uses a Clarkson integration algorithm for point dose calculation. To this end, 18 IMRT patient plans (186 individual beams) were created and used for RadCalc point dose calculations. In comparison with the primary treatment planning system (Monaco), mean point dose deviations of 0.0 ± 1.0% (n = 18) and 1.7 ± 12.4% (n = 186) were obtained on a per‐plan and per‐beam basis, respectively. The dose plane comparison functionality within RadCalc was found to be highly inaccurate, however, modest improvements could be made by artificially shifting jaws and multi leaf collimator positions to account for the dosimetric shift due to the magnetic field (67.3% vs 96.5% mean 5%/5 mm gamma pass rate).

## 1 INTRODUCTION

Image‐guidance in radiotherapy has significantly advanced the achievable conformality of treatment plans due to the improved ability to localize the target during each treatment fraction.^1^, ^2^ The availability of commercial technologies such as kV planar imaging, kV cone beam computed tomography (CBCT), CT‐on‐rails, online four‐dimensional‐CT/CBCT, stereotactic ultrasound, surface monitoring, and stereoscopic fiducial localization have significantly improved the clinician's ability to localize target and nontarget tissues. More recently, investment in MRI‐guided radiotherapy has grown rapidly due to the superior soft‐tissue contrast of the magnetic resonance imaging (MRI), the ability to perform repeated imaging without additional radiation dose, and the ability for continuous near real‐time intra‐fraction imaging.^3^, ^4^, ^5^ These advancements have created a number of paths for improved patient care including online adaptive planning, target tracking, and sophisticated gating techniques.^4^, ^6^, ^7^ It is expected that these developments will likely lead to margin reductions and related changes in practice patterns such as dose escalation and hypofractionation.

The first 1.5 T MRI‐equipped linear accelerator (Elekta Unity, Elekta AB, Stockholm, Sweden) was cleared by the U.S. Food and Drug Administration in December of 2018. Coupling a high‐magnetic field MRI with a linear accelerator introduces a number of commissioning and routine quality assurance challenges that are not associated with conventional linear accelerators. Notably, all equipment used for quality assurance must be MRI‐safe or MRI‐conditional, including daily quality assurance (QA) devices, IMRT QA devices, water tanks, and detectors used for output and beam scanning measurements. From a treatment planning perspective, conventional dose calculation algorithms are not suitable for the transport of charged particles in a magnetic field, so sophisticated algorithms that solve the linear Boltzmann transport equation must be utilized such as the stochastic Monte Carlo method or a deterministic grid‐based Boltzmann solver.^8^, ^9^, ^10^ Although these challenges have largely been addressed for primary treatment planning purposes, the need for independent dose verification prior to patient treatment remains.

The need for an efficient and accurate secondary monitor unit calculation is especially critical in the setting of online adaptive planning as a new treatment plan is generated for each fraction. Although a commercially available secondary dose calculator exists for the 0.35 T ViewRay MRIdian (ViewRay Inc., Oakwood, USA),^11^ there are currently only preliminary reports of purpose‐built software for the Elekta Unity.^12^ In this work, we explore the utility and performance of RadCalc software (Lifeline Software Inc., Tyler, USA), a widely used secondary point dose calculator, in conjunction with a newly commissioned Elekta Unity MRI‐linac. To our knowledge, there are no other reports in literature describing the commissioning and evaluation of this commercial product for use with the Elekta Unity MRI‐linac.

## 2 MATERIALS AND METHODS

### 2.1 Unity overview

The Elekta Unity MRI‐Linac is comprised of a Philips Marlin 1.5 T MRI (Philips Healthcare) and a standing‐wave linear accelerator. The linear accelerator produces a single 7‐MV flattening filter‐free photon energy with a maximum field‐size in the isocenter plane of 57.4 cm (crossplane) by 22.0 cm (inplane). Beam collimation consists of jaws (crossplane) and a 160‐leaf multi leaf collimator (MLC) (inplane), which has a leaf width of 7.175 mm in the isocenter plane. The Unity has a source to isocenter distance of 143.5 cm and an inner bore diameter of 70 cm. The gantry rotates with a speed of up to six rotations per minute and the collimator cannot be rotated. Currently, the system is only capable of delivering step‐and‐shoot intensity‐modulated radiation theraphy (IMRT) and three‐dimensional (3D) conformal treatments, however, no hardware limitations exist that would prevent future implementation of volumetric‐modulated arc therapy (VMAT) capabilities.

The University of Iowa Elekta Unity was calibrated to 1 cGy per MU in water at isocenter at a depth of 10 cm (133.5 cm SSD). This depth was chosen over the more conventional depth of d_max_ due to the fixed maximum dose rate of 425 MU/min for the Unity, regardless of calibration depth. Absolute output calibration of the Elekta Unity is complicated by nonstandard reference conditions, including but not limited to the presence of a magnetic field and a nonstandard source‐to‐axis distance. As such, orientation‐dependent correction factors for the ion chamber were required,^13^ and the TPR_20,10_ beam quality specifier was converted to PDD(10)_x_ for k_Q_ determination.^14^

### 2.2 Relative beam data

Beam data were collected during commissioning of the Unity MRI‐Linac in accordance with recommendations by Elekta and the requirements for beam modeling within the Monaco treatment planning system. As there is no commercially available 3D tank that is compatible with the Unity at the time of this writing, all depth dose and profile scans were acquired using a proprietary tank that is owned and provided by Elekta. Due to spatial constraints within the bore (and therefore within the 3D tank), the maximum scan depth was 12 cm when the gantry was set to 0^o^ (G0). With the gantry oriented to 90° or 270° (G90, G270), the maximum scan depth was 38 cm, however, the field size was limited to a maximum of 16 cm (crossplane) by 22 cm (inplane) in this orientation. As such, profile data for the field sizes of 2 × 2, 3 × 3, 5 × 5, 10 × 10, 15 × 15, 22 × 22, 40 × 22, 53.5 × 22, and 57.4 × 22 cm^2^ were acquired at depths of 1.3, 5, and 10 cm at the G0 orientation. Profile data for the field sizes of 2 × 2, 3 × 3, 5 × 5, 10 × 10, and 16 × 16 cm^2^ were acquired at depths of 1.3, 5, 10, 20, and 30 cm at the G270 orientation. PDDs were acquired to depths of 12 and 35 cm in the G0 and G270 orientations, respectively. Scans were acquired using a 0.07 cm^3^ ion chamber (PTW TN31021) and a microdiamond detector (PTW TN60019).

Due to the limited scan data for larger field sizes, the decision was made to commission RadCalc (v6.3, LifeLine Software, Inc.) using profiles and depth dose curves from the Monaco beam model rather than primary scan data. This was done following extensive validation of the Monaco model, including comparison against measured scan data, measured point dose data, and IMRT measurements. All model validation tests were within the relevant specified tolerances.^15^, ^16^, ^17^ The Monaco beam model was prepared for export by calculating dose for relevant open G0 field sizes on a water phantom in Monaco, and exporting sagittal and axial dose planes. A separate G180 plan was created in Monaco in order to calculate an open field for the largest field size (57.4 × 22), as this field‐size is prohibited in Monaco at G0 due to presence of the MRI cryostat pipe at the field periphery. All dose was computed with a specified Monte Carlo statistical uncertainty of 0.5% using a 2‐mm dose grid. A Python program was written to extract depth dose and profile data from these dose plane files. Extracted data was smoothed using Savitzky–Golay filtering to reduce noise from the Monaco Monte Carlo dose calculation and formatted for import into RadCalc. Percent depth dose (PDD) data were manually converted to tissue phantom ratio (TPR) data for RadCalc modeling, as the use of PDD data was found to lead to errors in phantom scatter factor lookup for nonreference condition SSDs due to inappropriate use of field‐size scaling. Asymmetric field‐sizes are not permitted in RadCalc beam modeling, so artificial inplane profiles for field‐sizes larger than 22 × 22 cm^2^ were generated from the corresponding crossplane profile.

**Figure 1:**
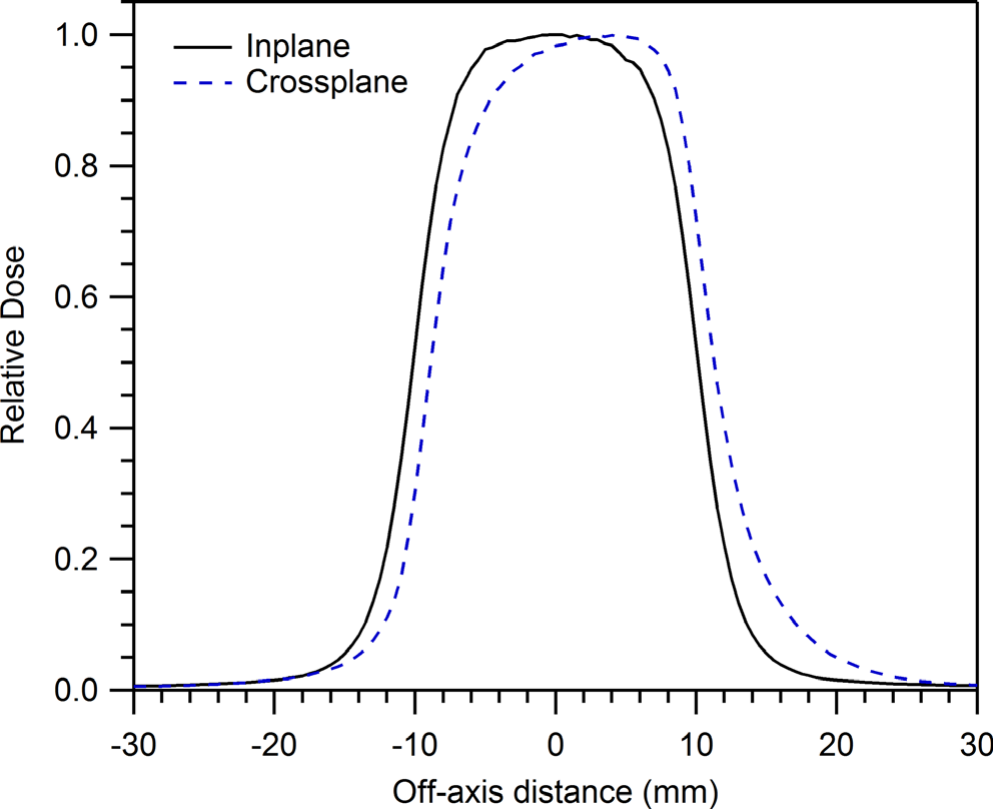
Impact of magnetic field on crossplane profile. Profiles acquired at G0, 1.3 cm depth in water using a microdiamond detector and a 2.0 cm × 2.0 cm field size.

Crossplane profiles for the Elekta Unity exhibit significant asymmetry due to the influence of the magnetic field on secondary electrons generated within the media. This effect has previously been described in literature,^18^, ^19^ and an example profile is shown in Fig. 1. RadCalc does not utilize user‐entered crossplane profiles unless a wedge is active, so this asymmetry cannot be accounted for. Even adding a wedge to the model and labeling the crossplane profiles as wedged fields does not achieve the desired result since the RadCalc fluence model assumes that the profiles should be centered about the central axis, and therefore the model’s fit to the data is inappropriate. For this reason, the method of enabling wedge profiles was not used in the RadCalc model generation.

### 2.3 Output factors

**Figure 2:**
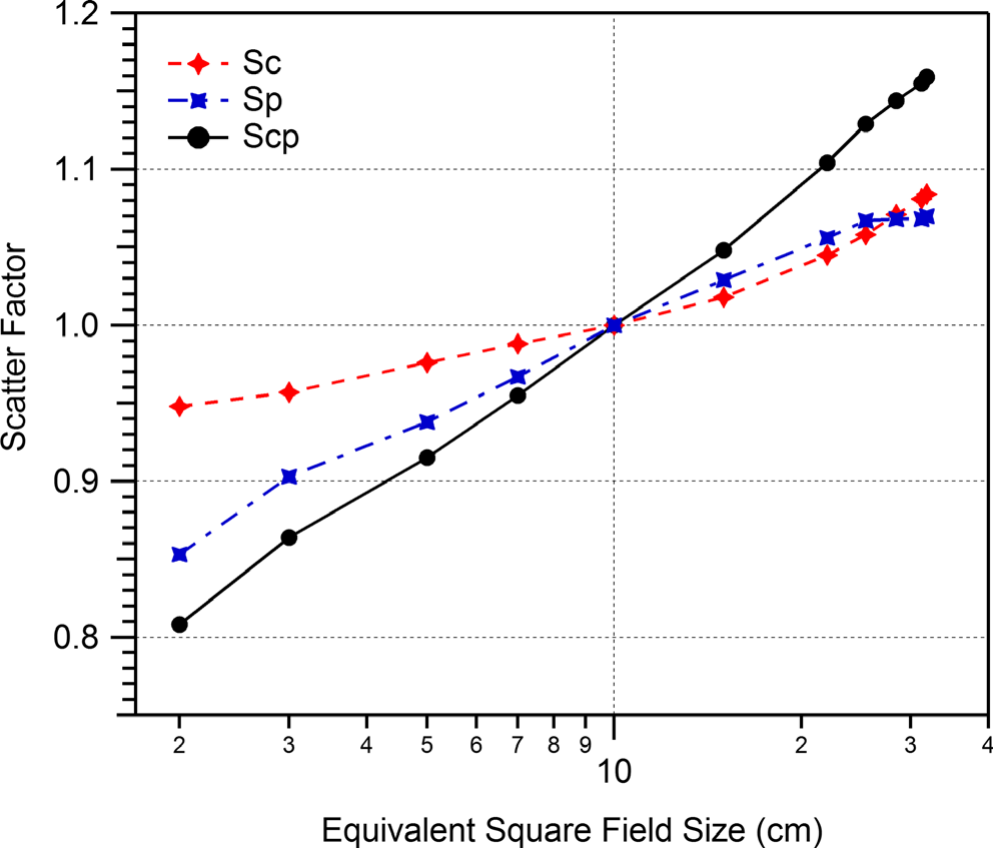
Measured (S_c_, S_cp_) and calculated (S_p_) output factors for the Elekta Unity.

Output factors were measured in accordance with recommendations by the AAPM Task Group 74.^20^ Briefly, total scatter factor data (S_cp_) were acquired in water with a microdiamond detector positioned at isocenter at 10 cm depth. Collimator scatter factors were measured using an ion chamber (0.125 cm^3^) in acrylic and brass miniphantoms for large and small field sizes, respectively. All final output factors were normalized to a 10 × 10 cm^2^ field size. Final collimator and phantom scatter factors (S_c_, S_p_) were imported into RadCalc. The final scatter factors used for RadCalc commissioning are shown in Fig. 2.

### 2.4 Model configuration

**Figure 3:**
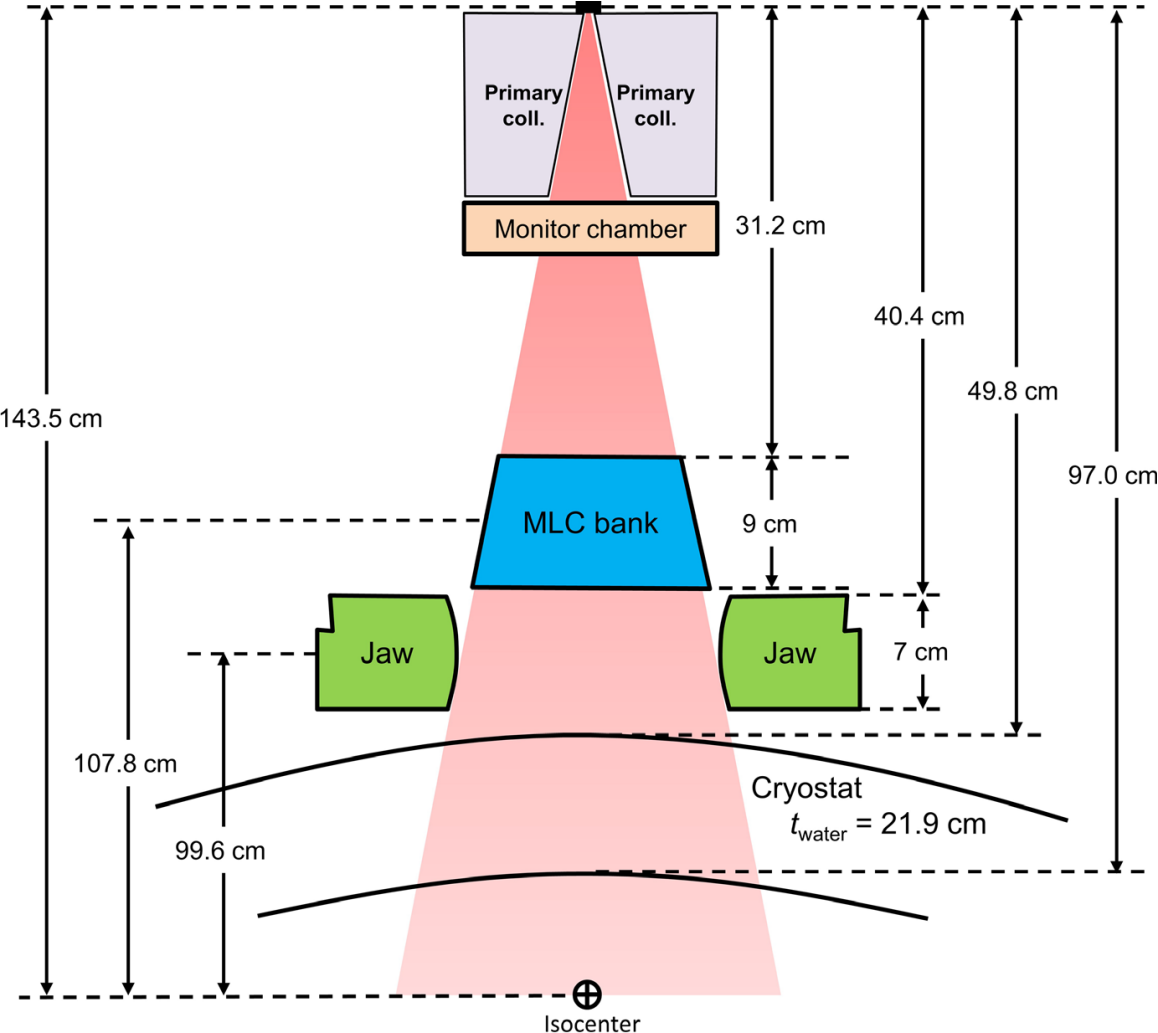
Schematic representation of the Elekta Unity radiation geometry.

Following creation of a treatment machine in RadCalc, dosimetry data were imported and a number of machine‐specific settings were configured. These settings are summarized in Table 1, and a schematic representation of the overall machine geometry is shown in Fig. 3. Several settings, including the maximum jaw position and source axis distance (SAD), had to be manually set in the machine.ini file, as these parameters were outside the bounds of acceptability by the graphical user interface. An output calibration of 0.99 cGy/MU was utilized due to the average difference between dose to medium across several tissue types and dose to water. For patient treatment plans, Monaco assumes a material based on the electron density of each voxel and subsequently computes dose to medium, whereas RadCalc assumes a specification of dose to water. This 1% correction factor to output represents the average of what is appropriate for varying tissues types. The Monaco treatment of dose calculation, and discrepancies with other methods, have been discussed by other authors in the literature.^21^, ^22^

**Table 1:** Specific settings used within RadCalc for modeling the Elekta Unity MRI‐linac.

Parameter	Setting
Source axis distance	143.5 cm
Source to block tray distance	48.1 cm
Couch vertical zero position	0. cm
Volume average dose options	5 mm search radius, automatically select best value
Clarkson radial sampling distance	0.500 cm
Clarkson angular sampling increment	5°
Clarkson radius used for primary dose	0.800 cm
Clarkson pixel size for intensity map	0.500 cm
Clarkson max angular step between control points	2.50°
Clarkson max leaf position change between control points	0.20 cm
Gantry minimum/maximum angle	0.0°/359.9°
Direction of positive gantry rotation	CW
Jaw transmission	0
Block transmission	0.035
MLC leaf transmission	0.007
Radiation/light field offset (MLC offset)	−0.130 cm
Energy value (MV)	7.00
Fluence mode	Non‐standard
Reference SSD	133.5 cm
Reference equivalent square	10.00 cm
RTP calibration factor (cGy/MU)	1.0000
D_max_ depth	1.30 cm
Reference depth	10.00 cm
Calibration @ reference	0.9900 cGy/MU
Front/back jaw min/max	−11 cm /11 cm
Left/right jaw min/max	−17.84 cm/28.70 cm
Source to top of front/back jaws	31.2 cm
Source to top of left/right jaws	40.4 cm
Select jaws that leaf motion is parallel to	F/B
Dose the MLC replace the F/B jaws	Yes
Source to top of MLC leaves	31.2 cm
Minimum leaf separation for closed leaves	0.6 cm
MLC front side name	Right
MLC back side name	Left
Swap leaves F/B	No
Swap leaves L/R	No
Leaves are in IEC convention	Yes
Leaf width/position	0.717 cm width, range from − 28.341 cm to + 28.341 cm
S_c_ correction factor	0.000
S_cp_ correction factor	0.000
Choose TPR or PDD for selected energy	TPR
Allow fluence corrections for selected machine	Yes

### 2.5 Point dose validation

To evaluate the performance of RadCalc as a secondary dose calculator for the Elekta Unity, 18 patient treatment plans averaging 10.3 IMRT beams per plan were created in Monaco. Treatment sites included pelvic nodes, prostate, brain, head and neck, liver, abdomen, and pancreas. For each treatment plan, a point of interest located within the center of the planning target volume (PTV) was selected in Monaco—this is the point at which dose is specified within the treatment plan file exported from Monaco. These radiation treatment plans were transferred to RadCalc without plan structures. The transfer of plan structures causes RadCalc to utilize its own internal region of interest (ROI) module for determination of effective depth for each control point. This improves the calculation accuracy for VMAT plans being transferred from other treatment planning systems, but this is unnecessary in this situation due to Monaco correctly specifying effective depths for each IMRT beam. Dose was calculated to the point of interest for each treatment plan, and statistics regarding agreement with Monaco were aggregated. In order to reduce the systematic deviation from dose calculated in Monaco, the leaf offset value (difference between nominal leaf position and radiation penumbra) was optimized to a final value of −1.3 mm as specified in Table 1.

In addition to dosimetric tests in the patient geometry, tests outlined by Smilowitz et al. in the AAPM Medical Physics Practice Guideline 5.a. (MPPG5a) were performed.^23^ These point‐dose tests were performed using a rectangular prism phantom of water with beams entering at orthogonal and oblique angles. For a detailed description of these tests, the reader is referred to MPPG5a.

### 2.6 Dose plane comparison

For this comparison, each of the test cases was projected onto a water phantom and the gantry angles were zeroed. This is required when using the RadCalc dose plane comparison functionality because RadCalc can only calculate dose planes which are orthogonal to the beam axis and Monaco can only calculate sagittal, coronal, and transverse dose planes. Coronal dose planes were exported from Monaco, and manually associated with the corresponding beam in each treatment plan in RadCalc. The three dose normalization options in RadCalc (avg, point, and max) were evaluated, and it was determined that normalizing to the average yielded the most consistent outcome. Analysis was performed using a gamma criterion of 5%/5 mm, a low dose threshold of 20%, and normalized using the average dose plane value. In an attempt to improve agreement between Monaco and RadCalc, a separate Unity model was created in RadCalc where the MLC leaves were shifted by 2 mm in the direction of the crossplane asymmetry. Additionally, a MATLAB program was written to adjust the jaw position of each control point within a given DICOM‐RT plan file. Both of these changes physically shifted the dose profile in the crossplane direction which mimicked the impact of the magnetic field. Changes to the radiation leaf offset were also investigated as a means to improve dose plane comparison performance.

## 3 RESULTS

### 3.1 Point dose validation and refinement

**Figure 4:**
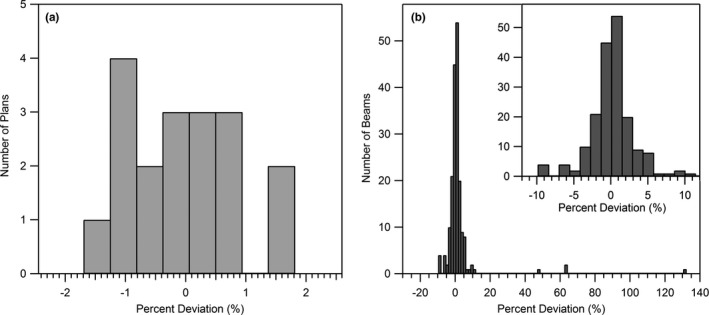
Histogram of (a) per‐plan percent deviations from Monaco, and (b) per‐beam percent deviations from Monaco. The four outlying per‐beam deviations represent a very small percentage of the total dose within their parent plan and were all outside of the primary field blocking.

The average per‐plan point dose deviation between RadCalc and Monaco was found to be 0.0 ± 1.0% (n = 18). The maximum, median, and minimum per‐plan deviations were 1.8%, −0.1%, and −1.7% respectively. The per‐beam deviation between RadCalc and Monaco was less precise, with an average of 1.7 ± 12.4% (n = 186). The maximum, median, and minimum per‐beam deviations were 131.7%, 0.1%, and −9.9% respectively. Point dose results for specific test cases are listed in Table 2. Point dose agreement distributions shown in Fig. 4 reveal an approximately normal underlying distribution for per‐beam comparisons. The distribution of per‐plan deviations is centered at about 0% and appears to be compactly supported in the domain of approximately −2% to +2%. Beams where the calculation point was near the field periphery had significantly worse point dose agreement with Monaco, ostensibly due to RadCalc’s inability to model the crossplane magnetic field effects. To illustrate this point, within Table 2 we have included the global percent deviation for outliers in parentheses.

**Table 2:** List of treatment plans evaluated in RadCalc, and their associated point dose deviations from Monaco. Percent differences are calculated as (D_RC_ − D_RTP_)/D_RTP_. Parentheses indicate the global percent deviation, obtained by multiplying the per‐beam percent difference by the fractional contribution of the individual beam against the sum of beams for the given plan. This demonstrates that beams with large percentage deviation represent a relatively minor portion of the overall point dose calculation.

Plan	Site	Overall agreement	Per‐beam maximum	Per‐beam median	Per‐beam minimum
1	Pelvic node	−0.7%	63.5% (0.74%)	0.1%	−9.0%
2	Pelvic node	−0.9%	60.7% (0.81%)	0.1%	−9.6%
3	Brain	0.9%	47.3% (0.80%)	0.2%	−8.9%
4	Brain	−0.1%	10.1%	0.2%	−3.0%
5	Prostate	−1.0%	0.8%	−0.7%	−6.4%
6	Prostate	−1.7%	2.5%	−0.3%	−9.9%
7	Liver	0.2%	5.0%	0.1%	−6.4%
8	Liver	−0.8%	131.7% (0.31%)	0.5%	−6.4%
9	Abdomen	−0.2%	2.5%	0.1%	−5.1%
10	Abdomen	1.5%	5.0%	0.7%	−2.5%
11	Pancreas	−0.9%	1.7%	0.0%	−6.6%
12	Pancreas	0.9%	8.8%	0.0%	−1.9%
13	Pancreas	0.2%	0.0%	0.2%	−9.6%
14	Pancreas	1.8%	0.0%	2.1%	−1.7%
15	Head and neck	0.8%	5.4%	1.2%	−4.2%
16	Pelvic node	0.3%	3.7%	0.2%	−3.9%
17	Prostate	−0.1%	2.8%	0.2%	−6.2%
18	Liver	−0.9%	3.5%	−0.9%	−3.5%

**Figure 5:**
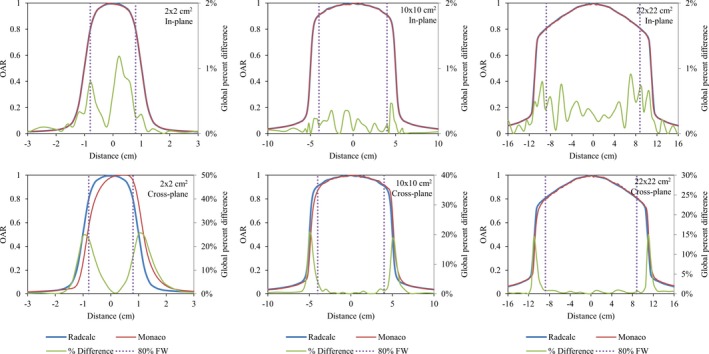
Comparison of RadCalc‐modeled profiles against baseline Monaco data that were used for commissioning of RadCalc. All inplane profiles were found to meet the MPPG5a‐specified deviation tolerance of 2%, however, crossplane profiles failed to meet this standard due to magnetic field effects. For larger field sizes (10 × 10 cm^2^ and 22 × 22 cm^2^) within the central 80% of the field, crossplane agreement was found to be within 2%.

Results from MPPG5a dosimetric evaluation are summarized in Table 3. All tests met the performance levels described in MPPG5a with the exception of crossplane profile agreement between RadCalc and commissioning data (Fig. 5), and point dose agreement for the large MLC‐shaped field with extensive blocking (MPPG5a test 5.5). Disagreement between crossplane profiles is expected due to magnetic field effects. Disagreement for the large MLC‐shaped field (e.g., “Mantle”‐shaped) may be due to uncertainty in the Monaco Monte Carlo calculation in addition to the modified Clarkson integral calculation methodology employed by RadCalc.

**Table 3:** Summary of MPPG5a^23^‐ recommended tests that were performed for RadCalc. Test 5.1 and 5.9 are not applicable in this situation, as the Unity does not have a wedge, and there are no separate dosimetry modules within RadCalc.

Test	Description	Result	Tolerance
MPPG5a 5.1	Dose distribution in planning module vs physics module	Not applicable	–
MPPG5a 5.2	Dose in test plan vs reference calibration condition	−0.3%	±0.5%
MPPG5a 5.3	TPS data vs. commissioning data	See Fig. 5	±2%
MPPG5a 5.4	Small MLC‐shaped field	−0.3%	±2%
MPPG5a 5.5	Large MLC‐shaped field with extensive blocking	−2.6%	±2%
MPPG5a 5.6	Off‐axis MLC shaped field	3.7%	±5%
MPPG5a 5.7	Asymmetric field at minimal anticipated SSD	−2.3%	±5%
MPPG5a 5.8	10 × 10 cm^2^ field at oblique incidence (>20°)	−2.1%	±5%
MPPG5a 5.9	Large field for each nonphysical wedge angle	Not applicable	–

### 3.2 Dose plane comparisons

**Figure 6:**
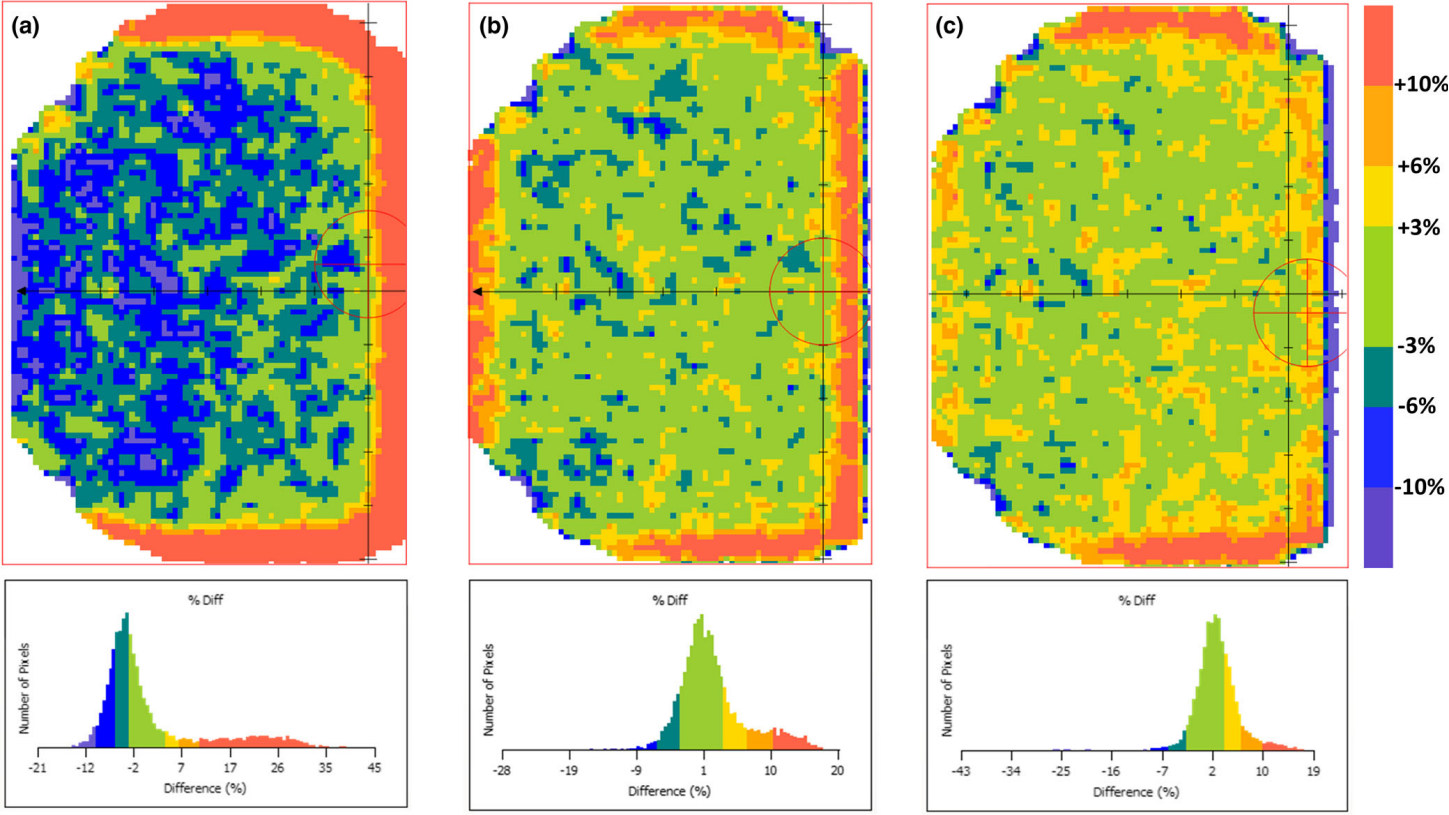
Dose plane comparison using the nominal RadCalc model (a); after applying a 2‐mm shift to the multi leaf collimator (MLC) and jaw positions and increasing leaf offset to +2 mm (b); and after applying a 2‐mm shift to the MLC and jaw positions, increasing the leaf offset to +2 mm, and moving the jaws outward by 1.5 mm each (c).

Comparison of dose planes calculated in Monaco and RadCalc revealed significant discrepancies. An average 5%/5 mm gamma pass‐rate of approximately 80% was observed in beams examined (n = 136), which motivated investigation into methods for improving dose plane comparison performance. As shown in Fig. 6(a), examination of the dose plane peripheries revealed the impact of the magnetic field‐induced crossplane asymmetry. The separate beam model with the 2‐mm shifted MLCs and associated jaw positions was found to improve agreement in the crossplane direction, however, the negative leaf offset that was required to reduce systematic point dose differences appeared to have a deleterious effect near the in‐plane penumbra. To address this, the leaf offset was modified in the new Unity model from −1.3 mm to +2.0 mm.

Systematically moving the leaves and jaws, and increasing the leaf offset value substantially improved dose plane agreement between RadCalc and Monaco [Figs. 6(a) and 6(b)]. It was noted that although a 2‐mm shift of the jaws was sufficient to symmetrize the lateral periphery of the percent difference map, RadCalc still underestimated peripheral dose in the lateral direction. To improve this behavior, the jaws were moved a further 1.5 mm outward (cumulative 0.5 shift for X1, 3.5 mm shift for X2). This also improved dose plane performance, as shown in Fig. 6(c).

In summary, the following changes were made to create a “dose plane specific model”: MLC leaves moved in the direction of the crossplane shift by 2 mm; X1 jaw moved 0.5 mm in the direction of the crossplane shift; X2 jaw moved 3.5 mm in the direction of the crossplane shift; radiation leaf offset increased from −1.3 mm to +2.0 mm. The degree to which these changes improved dose plane comparison performance, as measured by gamma analysis (5%/5mm), is exemplified by the patient plan summarized in Table 4. When normalizing to the dose plane average, the percent of points passing the gamma criteria increased from an average of 67.3% to 96.5%. It is worth noting that this jaw shift correction does not account for the depth‐dependent nature of the crossplane profile shift.^12^

**Table 4:** Dose plane comparison gamma analysis (5%/5 mm) before and after implementation of the dose plane specific model. Significant improvement using the dose plane specific model is seen over the standard model for all normalization options (calculation point, maximum, or average).

Beam	Standard model			Dose plane‐specific model		
	Calc	Max	Avg	Calc	Max	Avg
1	89.5%	54.9%	59.1%	98.3%	54.4%	95.3%
2	34.3%	54.2%	83.9%	98.7%	93.3%	94.8%
3	46.2%	72.8%	57.7%	58.0%	57.3%	95.2%
4	99.1%	40.1%	58.6%	99.7%	71.5%	99.8%
5	98.9%	51.0%	83.6%	99.7%	95.7%	85.2%
6	68.2%	60.4%	64.9%	82.2%	59.9%	96.8%
7	86.4%	83.2%	88.5%	99.2%	98.3%	98.6%
8	98.9%	84.2%	75.9%	99.3%	96.0%	99.0%
9	91.0%	89.5%	58.5%	88.7%	74.1%	98.3%
10	98.7%	62.1%	51.9%	99.0%	80.4%	98.9%
11	96.5%	83.4%	58.0%	96.3%	69.6%	99.1%
Avg	82.5%	66.9%	67.3%	92.6%	77.3%	96.5%

## 4 DISCUSSION

To our knowledge this work comprises the first reported commissioning experience of a commercially available secondary point dose calculator used with the Elekta Unity. As there are currently no commercially available secondary dose verification products that account for impacts of the magnetic field, we chose to assess the viability of utilizing RadCalc for this purpose. According to a survey conducted by AAPM Task Group 219, approximately 40% of all radiation oncology centers in the U.S. utilize RadCalc for purposes of monitor unit verification.^24^ We have described a number of model parameters, many obtained directly from Elekta, that may be useful to other investigators and clinicians in commissioning their own secondary dose calculator for the Elekta Unity.

The RadCalc algorithm utilizes a modified Clarkson integration technique for IMRT dose calculation. Several assumptions are made with this approach, namely flat patient entrance surface, homogeneous tissue surrounding the calculation point, and no magnetic field present. The modified Clarkson integration method relies on azimuthal symmetry of beam scattering, which is an assumption that does not hold true for the presence a magnetic field that is nonparallel to the central beam axis. For this reason, it was expected that significant deviations from the primary treatment planning system would be observed during the RadCalc commissioning and validation process. A number of improvements could potentially be made to the modified Clarkson integration method to improve its applicability to the MRI‐linac, as exemplified by the recent work of Chen et al.^12^ In their work, they describe implementation of a modified Clarkson integration method for the Elekta Unity whereby an angle‐ and depth‐dependent positional correction factor is applied for each integration sector. Although this approach ignores changes to the profile shape, the first‐order magnetic field effect is mitigated. The relative precision described by Chen et al.—a standard deviation of 4.6%—is significant improvement over the per‐beam standard deviation of 12.4% that was observed for RadCalc in this work. With that said, in the case of calculation points near tissue density heterogeneities, or for oblique surface entries, it is likely that both the RadCalc and Chen et al. methods will perform poorly in comparison with more sophisticated dose calculation techniques. Although the Chen et al. method likely performs better than RadCalc for MRI‐Linac point dose calculations, it is not currently widely available for clinical implementation.

Per‐plan point dose comparisons were found to agree reasonably well with Monaco in this work. Given that all of the 18 test plans have overall deviations between −1.7% and +1.8% for the commissioned model, a tolerance level of 3% and an action level of 5% was implemented clinically. Individual beams in a clinical plan with a deviation greater than ~8% are manually reviewed. If the control point is near the field periphery, or outside the field entirely, no corrective action is warranted. These tolerances may be made tighter as additional clinical experience is gained. To achieve this level of agreement, a search radius of 5 mm was implemented in RadCalc, which will likely reduce the overall sensitivity to error detection. Regardless, these results are likely adequate for detection of gross errors prior to patient treatment. Currently, all adaptive plans created on the Elekta Unity are being measured using a conventional IMRT QA device following patient treatment. These patient‐ and plan‐specific measurements are likely a much more sensitive tool for error detection. No patients with lung targets were utilized in this work, which is likely a situation where RadCalc would perform worse than what is presented here.

The dose plane comparison functionality within RadCalc did not appear to be particularly reliable for this application without substantial modification to the beam model and DICOM‐RT plan files. It is worth noting that routine manual editing of treatment plan files introduces a level of clinical risk and time inefficiency that must be weighed against the potential benefit from improved dose plane evaluation capabilities. Additionally, the 5%/5 mm gamma analysis used herein is significantly weaker than literature recommendations (typically 2%/2 mm), however, performing the analysis with tighter tolerances produced gamma pass rates that were sufficiently low as to not provide any useful information. It is our opinion that the two‐dimensional dose plane comparison tool in RadCalc is not currently suitable for use with the Elekta Unity.

Future work should include careful evaluation of the sensitivity of RadCalc for error detection. Comparison of results obtained with RadCalc against future purpose‐built secondary dose calculations will inform of their superiority with regard to precision and accuracy of dose calculation.

## 5 CONCLUSIONS

We have commissioned RadCalc as a secondary dose calculation tool for use with the Elekta Unity MRI‐linac. The point dose calculation was found to produce agreements to the primary Monaco Monte Carlo dose calculation of 0.0 ± 1.0% (n = 18) for per‐plan evaluations and 1.7 ± 12.4% (n = 186) for per‐beam evaluations. It was also found that the planar dose calculation was not accurate enough for clinical use using the standard model. Although significant improvement in agreement was obtained through modification of the standard model, it was determined that manually editing each treatment plan introduced unacceptable risk and increased the verification time. Future commercial dose calculation platforms that explicitly account for magnetic field effects will likely improve upon these results.

## CONFLICT OF INTEREST

No conflict of interest.
